# Effect of Unripe Banana Flour on Gut-Derived Uremic Toxins in Individuals Undergoing Peritoneal Dialysis: A Randomized, Double-Blind, Placebo-Controlled, Crossover Trial

**DOI:** 10.3390/nu13020646

**Published:** 2021-02-17

**Authors:** Laila Santos de Andrade, Fabiana Andréa Hoffmann Sardá, Natalia Barros Ferreira Pereira, Renata Rodrigues Teixeira, Silvia Daniéle Rodrigues, Jordana Dinorá de Lima, Maria Aparecida Dalboni, Danilo Takashi Aoike, Lia Sumie Nakao, Lilian Cuppari

**Affiliations:** 1 Nutrition Program, Universidade Federal de São Paulo—UNIFESP, São Paulo 05508-000, Brazil; laila.andrade27@yahoo.com.br (L.S.d.A.); nataliabfp@gmail.com (N.B.F.P.); renatart2@msn.com (R.R.T.); 2 Department of Food and Experimental Nutrition, Faculty of Pharmaceutical Sciences, Universidade de São Paulo—USP, São Paulo 05508-000, Brazil; fahsarda@alumni.usp.br; 3 APC Microbiome Ireland, University College Cork, Cork T12 K8AF, Ireland; 4 Department of Basic Pathology, Universidade Federal do Paraná—UFPR, Curitiba 81531-980, Brazil; silviahellfire@gmail.com (S.D.R.); lia.nakao@ufpr.br (L.S.N.); 5 Department of Cell Biology, Universidade Federal do Paraná—UFPR, Curitiba 81531-980, Brazil; jordanabioufpr@gmail.com; 6 Post-Graduate Program in Medicine, Universidade Nove de Julho—UNINOVE, São Paulo 01504-001, Brazil; mariadalboni@gmail.com; 7 Division of Nephrology, Universidade Federal de São Paulo—UNIFESP, Rua Botucatu, 720/740, São Paulo 04023-062, Brazil; dtaoike@gmail.com; 8 Dialysis Department, Hospital do Rim—Fundação Oswaldo Ramos, São Paulo 04038-002, Brazil

**Keywords:** unripe banana flour, uremic toxins, gut, prebiotic, chronic kidney disease

## Abstract

In chronic kidney disease (CKD), the accumulation of gut-derived metabolites, such as indoxyl sulfate (IS), p-cresyl sulfate (pCS), and indole 3-acetic acid (IAA), has been associated with the burden of the disease. In this context, prebiotics emerge as a strategy to mitigate the accumulation of such compounds, by modulating the gut microbiota and production of their metabolites. The aim of this study was to evaluate the effect of unripe banana flour (UBF—48% resistant starch, a prebiotic) on serum concentrations of IS, pCS, and IAA in individuals undergoing peritoneal dialysis (PD). A randomized, double-blind, placebo-controlled, crossover trial was conducted. Forty-three individuals on PD were randomized to sequential treatment with UBF (21 g/day) and placebo (waxy corn starch—12 g/day) for 4 weeks, or vice versa (4-week washout). The primary outcomes were total and free serum levels of IS, pCS, and IAA. Secondary outcomes were 24 h urine excretion and dialysis removal of IS, pCS, and IAA, serum inflammatory markers [high-sensitivity C-reactive protein (hsCRP), interleukin-6 (IL-6), interleukin-10 (IL-10), and tumor necrosis factor-α (TNF-α)], serum lipopolysaccharide LPS, and dietary intake. Of the 43 individuals randomized, 26 completed the follow-up (age = 55 ± 12 years; 53.8% men). UBF did not promote changes in serum levels of IS (*p* = 0.70), pCS (*p* = 0.70), and IAA (*p* = 0.74). Total serum IS reduction was observed in a subgroup of participants (*n* = 11; placebo: median 79.5 μmol/L (31–142) versus UBF: 62.5 μmol/L (31–133), *p* = 0.009) who had a daily UBF intake closer to that proposed in the study. No changes were observed in other secondary outcomes. UBF did not promote changes in serum levels of IS or pCS and IAA; a decrease in IS was only found in the subgroup of participants who were able to take 21g/day of the UBF.

## 1. Introduction

Numerous metabolites that normally are part of the mammalian serum metabolome are derived from the metabolism of the gut microbiota [1,2]. Among them, indoxyl sulfate (IS), indole 3-acetic acid (IAA), and p-cresyl sulfate (pCS) are generated from the bacterial fermentation of amino acids tryptophan and tyrosine. In the distal colon, the fermentation of tryptophan results in indole and IAA and the result of tyrosine fermentation is p-cresol. By sulfate conjugation in the liver and in colonic mucosa, p-cresol and indole are converted into pCS and IS, respectively [3,4]. In circulation, more than 90% of these compounds are bound to proteins and are excreted by the kidneys [5,6]. In chronic kidney disease (CKD), due to gradual loss of kidney function, several compounds are retained, including those derived from the gut microbiome. Serum concentration of these compounds increases gradually as renal function decreases, reaching the highest level in dialysis [7,8]. Since they are bounded to serum protein, their dialytic clearance is low [8,9]. Accumulation of these uremic toxins has been associated with the progression of CKD [10], inflammation [11,12], cardiovascular disease [13,14], and mortality from cardiovascular disease and all causes in individuals with CKD [15,16,17].

Studies have shown that CKD is associated with altered colonic microbiota composition [18,19,20,21]. Among these changes, increased bacterial families with producers of indole and p-cresol were observed, suggesting a potentially upregulated microbiota in the production of these metabolites [19]. However, changes in the composition of the microbiota do not necessarily implicate alterations in microbial metabolism [22]. Recently, in a well-designed study, it was demonstrated that the gut microbiota production of these metabolites does not change as renal function decreases [23]. 

Diet seems to be the main determinant of gut microbial metabolism [24]. The type and amount of substrate that reach the human colon are key modulators of bacterial composition and metabolism. A greater availability of carbohydrates in relation to nitrogen compounds favors the growth of bacteria that preferentially ferment carbohydrates over the fermentation of nitrogenous compounds, resulting in beneficial changes in the gut microbiota’s composition and in the pattern of metabolites produced [25,26,27]. A markedly lower production of IS and pCS was associated with lower protein and higher fiber intake in vegetarian individuals [28]. In individuals with CKD, a positive correlation between dietary protein–fiber ratio and serum levels of IS and pCS was observed [29]. Due to dietary restriction on fruit, vegetables, and legumes to control hyperkalemia, low fiber intake is frequent among individuals with CKD [30,31], which may favor the fermentation of amino acids and consequently, contribute to the accumulation of IS and pCS.

In this context, prebiotics emerge as a strategy to mitigate the accumulation of these compounds, by modulating the gut microbiota and production of metabolites towards lower amino acids fermentation. Prebiotics are substrates selectively utilized by host microorganisms, providing health benefits. Among them are fermentable dietary fibers, such as inulin, fructo-oligosaccharides (FOS), galacto-oligosaccharides (GOS), and resistant starch (RS). When these undigested carbohydrates reach the colon, they are fermented by specific colon bacteria, which leads to changes in the composition and metabolic activity of the gut microbiota, benefiting the health of the host [32,33]. To date, there is a paucity of well-designed studies about prebiotics’ impact on serum levels of gut-derived uremic toxins in CKD. Among the studies conducted so far, the impact on pCS levels is more common than on IS. Recently, RS has emerged as a possibility to reduce the microbial production of IS [34,35]. In two studies with patients undergoing hemodialysis, the use of RS resulted in a reduction in IS serum levels [34,36]. Among foods with a significant amount of RS, the flour obtained from unripe banana, which contains about 50% RS, may represent a potential option to provide large amounts of prebiotic [37]. Adding to that, the great availability of bananas in Brazil and the low cost of flour production mean unripe banana flour (UBF) may be an attractive foodstuff to be used in the CKD setting [38].

In this sense, we tested for the first time whether providing UBF to individuals with CKD undergoing peritoneal dialysis would reduce the serum gut-derived uremic toxins (IS, pCS, and IAA).

## 2. Materials and Methods

This is a randomized, double-blind, placebo-controlled, crossover trial. The study was approved by the Ethics Committee of Universidade Federal de São Paulo (UNIFESP, São Paulo, Brazil) and registered at the Brazilian Clinical Trials Registry (www.ensaiosclinicos.gov.br, ID number: RBR-4xxwwj). Written informed consent was obtained from each participant.

### 2.1. Study Population

Patients undergoing automated peritoneal dialysis (APD) from Fundação Oswaldo Ramos outpatient clinic (São Paulo, Brazil), aged 18–80 years, with a dialysis vintage of at least three months and adherent to dialysis treatment were invited to participate in the study. The exclusion criteria were the use of prebiotics, probiotics, synbiotics, and antibiotics one month before the beginning of the study, the presence of inflammatory bowel diseases, stomach, or bowel resection, liver cirrhosis, cancer, human immunodeficiency virus, peritonitis in the last month, pregnancy, and breastfeeding.

Participants were discontinued from the study if they presented any gastrointestinal intolerance even after dose adjustments, were hospitalized, started antibiotic therapy, underwent kidney transplant, or wished to be withdrawn.

### 2.2. Intervention

The UBF was prepared in the Food Research Center (FoRC) of the Universidade de São Paulo (USP) with the unripe banana pulp, *Musa acuminata* (group AAA), subgroup Cavendish, at the first stage of maturation (unripe), not subjected to a maturation chamber, and purchased at a local market. The production of UBF was performed in accordance with the patented process (PI 0705778-4) proposed by Tribess et al. [38] to preserve resistant starch (RS). RS and total starch (TS) present in the UBF were quantified by the AOAC 2002.02 method [39] and the method described by Cordenunsi and Lajolo [40], respectively. The flour produced contained approximately 48% resistant starch. In addition to resistant starch, UBF has other types of fibers (about 7%) and small amounts of phytosterols and polyphenols [37]. Regarding the potassium content, in 100 g of UBF, there are approximately 1133 mg of potassium. Waxy corn starch (Amioca), supplied by Ingredion Incorporated (Ingredion Brasil Ingredientes Industriais Ltda., São Paulo, Brazil), was used as placebo.

Supplements were packaged in identical sachets and identified by the numbers 225 and 653 in a handling pharmacy, under the responsibility of a pharmacist (Magister Pharmacy, Sao Paulo, Brazil). Each UBF sachet contained 10.5 g of flour (5 g of RS) and the placebo sachets contained 6 g waxy corn starch. The amount of placebo was defined in order to provide a similar amount of energy in relation to UBF (21 kcal/sachets).

The initial dose of both interventions was one sachet per day. After the three initial days, in the absence of adverse effects, participants were advised to double the daily amount of each supplement. In the presence of difficulties to take the proposed final dose (two sachets daily), such as any adverse gastrointestinal symptom, the participant was maintained in the study with the tolerable dose. Participants were advised to take the supplements during meals, mixed in any drink or food at low to moderate temperature in order to preserve the resistant starch.

### 2.3. Study Protocol

**Figure 1:**
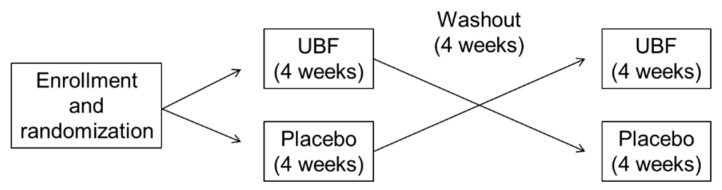
Scheme of the study protocol. UBF—unripe banana flour.

The participants were assigned to sequential treatment with UBF and placebo, or vice versa, after a blocked randomization procedure using a random block of 4 participants in a 1:1 ratio. A computerized random list was generated by an independent researcher. The list was handed over to another independent researcher who was responsible for separating and allocating supplements to participants. The duration of each intervention was 4 weeks with a washout period (4 weeks) between them. Before and after each intervention, venous blood, 24 h dialysate, and 24 h urine of participants with daily urinary volume ≥200 mL were collected. Figure 1 shows the study protocol.

The majority of the participants had been previously advised by a renal dietitian, according to the nutritional guidelines for patients undergoing peritoneal dialysis [41,42], in the outpatient clinic routine. During each intervention, the participants were advised to maintain a stable dietary pattern and not to take prebiotics, probiotics, or synbiotics. If necessary, dietary adjustments were advised or reinforced during the follow-up. The sachets were delivered to the participants during their monthly visit to the outpatient clinic. In the first week of each intervention, an independent researcher, through phone calls, elucidated possible doubts regarding the use of the supplement and, when necessary, adjusted the dose with the participant, encouraging the regular use of the supplement. To evaluate adherence, sachets were counted at the end of each arm of the study.

### 2.4. Outcome Measures

The primary outcomes were serum levels of IS, pCS, and IAA. The secondary outcomes were 24 h urine excretion and dialysis removal of IS, pCS, and IAA, serum inflammatory markers (high-sensitivity C-reactive protein (hsCRP), interleukin-6 (IL-6), interleukin-10 (IL-10), and tumor necrosis factor-α (TNF-α)), serum lipopolysaccharide (LPS), dietary intake, and gastrointestinal symptoms.

### 2.5. Demographic, Clinical, and Biochemical Data

Demographic and clinical data were collected from medical records. Venous blood samples were collected in the morning and under fasting conditions (12 hours). Aliquots were immediately centrifuged and the serum was stored at −80 °C. Aliquots of urine and dialysate were also stored at −80 °C.

Serum, urinary, and dialysate concentrations of IS, pCS, and IAA were quantified by high-performance liquid chromatography (HPLC) with fluorescent detection. Samples were diluted with the exception of dialysate, heated at 95 °C for 30 minutes and centrifuged at 13,000 rpm for 10 minutes at room temperature. Then, the samples were cooled on ice for 10 minutes, filtered through 0.22 mm filter, and injected into the system of HPLC. To quantify the free fractions, the serum samples without dilution were only centrifuged and filtered (0.22 mm). The chromatographic system was the one described by Rodrigues et al. [43]. During the run, fluorescence wavelengths varied: λ_exc_ = 283 nm and λ_em_ = 380 nm for IS and IAA and λ_exc_ = 265 nm and λ_em_ = 290 nm for pCS.

Serum IL-6, IL-10, and TNF-α were measured using a kit of 3-cytokine Milliplex MAP Human Cytokine/Chemokine Magnetic Bead Panel (EMD Millipore Corp., Billerica, MA, USA) following the specific protocol provided by the manufacturer. Serum LPS was determined using the Hycult Biotech Limulus Amebocyte Lysate assay (Hycult Biotech, Uden, The Netherlands). Serum levels of urea, creatinine, sodium, potassium, ionized calcium, phosphorus, glucose, albumin, glycated hemoglobin, and high-sensitivity C-reactive protein were measured by standard techniques. Residual renal function (RRF) was calculated by the mean of creatinine and urea clearance corrected for body surface area [44]. Dialysis efficiency (Kt/V) was calculated using the equation recommended by the Clinical Practice Guidelines for Peritoneal Dialysis Adequacy [45].

### 2.6. Dietary Intake

A food record of three alternate days, including the day before blood collection, was used to estimate energy, protein, and fiber intake. Participants completed the records at the beginning and at the end of each intervention. All records were checked in a face-to-face interview by the same dietitian, using food models and household utensils to improve the accuracy of the recorded data. The software Nutwin^®^ (UNIFESP, São Paulo, Brazil) was used to calculate the average nutrient intake.

Protein intake was also estimated by the protein equivalent of nitrogen appearance (PNA), normalized by desirable or adjusted body weight [41].

### 2.7. Gastrointestinal Symptoms

Gastrointestinal symptoms were monitored using the Gastrointestinal Symptom Rating Scale (GSRS) questionnaire, translated and validated into Brazilian Portuguese. This questionnaire consists of 15 questions comprising the following symptoms: abdominal pain, reflux, nausea, borborygmi, abdominal distension, belching, flatulence, diarrhea, and constipation. The intensity and/or frequency of each symptom are scored according to a seven-level Likert scale. The total GSRS score ranged from 15 to 105 points. The higher the score, the higher the intensity and/or frequency of the symptom [46].

### 2.8. Statistical Analysis

The sample size was calculated using, as a reference, the study of Sirich et al. [34]. A total of 35 participants were estimated to detect a reduction of 17% in serum total IS, with a power of 80% and an error level of 0.05. Allowing a dropout rate of 20%, 43 participants were enrolled in the study. GPower software, version 3.1.2 (Franz Faul, University of Kiel, Kiel, Germany) was used to calculate the sample size.

The data are presented as the mean and standard deviation (SD), median and interquartile range, or frequency and percentage, as appropriate. Student’s t-test or Mann–Whitney test and Pearson's Chi-square or Fisher’s exact test were used to compare the baseline characteristics between the patients who discontinued and patients who completed the follow-up. Generalized estimating equations (GEE) were used to analyze the effect of intervention on the variables. Statistical significance was established at p values < 0.05. All statistical analyses were performed using the SPSS^®^ software version 18.0 for Windows (SPSS Inc., Chicago, IL, USA).

## 3. Results

### 3.1. Study Participants

**Figure 2:**
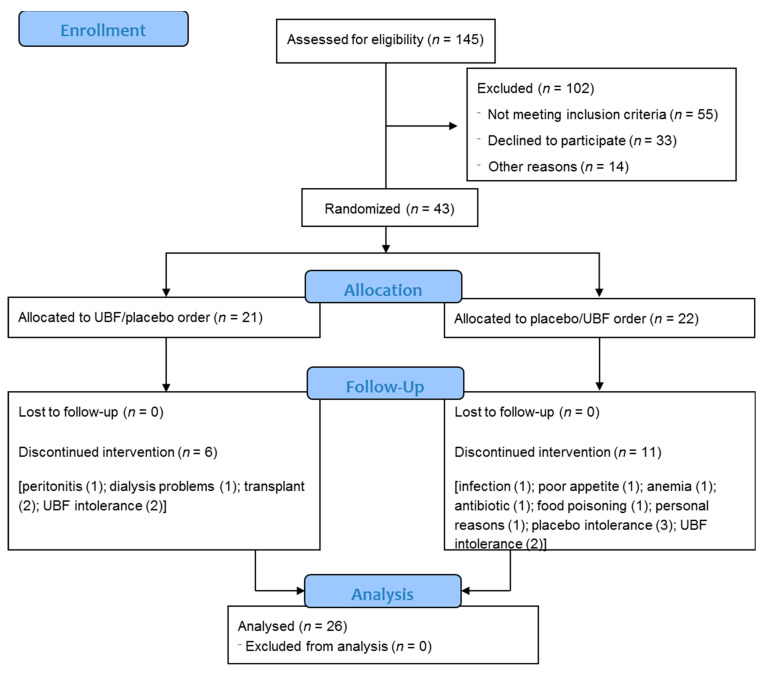
Consolidated Standards of Reporting Trials (CONSORT) flow diagram. UBF—unripe banana flour.

Participants were recruited between May 2018 and September 2018 from a single peritoneal dialysis center. Forty-three participants were recruited and randomly allocated to sequential treatment with UBF and placebo, or vice versa. Twenty-six participants concluded both arms of the trial, as depicted in the Consolidated Standards of Reporting Trials (CONSORT) flow diagram (Figure 2).

In the study population, the causes of CKD were diabetic nephropathy (19.2%), hypertensive nephropathy (3.8%), glomerulonephritis (7.7%), polycystic kidney disease (15.4%), other causes (11.5%), and unknown causes (42.3%). The majority of participants were men (53.8%), 34.6% had diabetes, 84.6% had residual diuresis, and 92.3% were undergoing nocturnal intermittent peritoneal dialysis (NIPD). The most common dialysis prescription was 5 cycles per 9 hour cycling session with 10L of total volume infusion using a glucose-based solution. The participants were on antihypertensive medication (96%), insulin (31%), statins (73%), and phosphate binders (65.5%). Table 1 shows that except for the GSRS score that was higher in patients who discontinued the study, no other differences were observed between the group of participants who completed the study and the group of participants who discontinued the study.

### 3.2. Adherence, Tolerance, and Gastrointestinal Symptoms

Seven patients (26.9%) in the UBF arm and four patients (15.4%) in the placebo arm did not tolerate taking two sachets per day; therefore, they were maintained with the tolerable dose (one sachet/day). The reasons for the intolerance to the initially proposed dose were the large volume of flour (placebo: three patients; UBF: four patients), intolerable increase in flatulence (UBF: two patients), and hardening of stools (placebo: one patient; UBF: one patient). Taking these dose adjustments into account, the overall adherence was good in both arms (placebo: median adherence 86.7% (67.3–92.6) versus UBF: 83% (64.6–94.5), *p* = 0.67). The median daily intake of placebo was 9.6 g (6.6–10.4) and UBF 14 g (11.4–18). Therefore, the daily intake of resistant starch in the UBF arm was about 6.8 ± 2.1 g (48% of UBF).

With regard to gastrointestinal symptoms, no change was observed in the GSRS score between the interventions (*p* = 0.30).

### 3.3. Laboratory Parameters and Dietary Intake

As shown in Table 2, no differences were observed in the majority of laboratory parameters and dietary intake between the arms. A slight increase in the serum levels of IL-6 was observed after supplementation with UBF. At baseline, the protein:fiber ratio was higher in the UBF compared to the placebo.

### 3.4. Uremic Toxins

As can be seen in Table 3, UBF did not promote changes in both total and free serum levels of IS, pCS, and IAA. The excretion of total IS, pCS, and IAA through 24 h urine and dialysate was also not different between the arms during the follow-up.

### 3.5. Subgroup Based on Adherence to the Supplement

**Figure 3:**
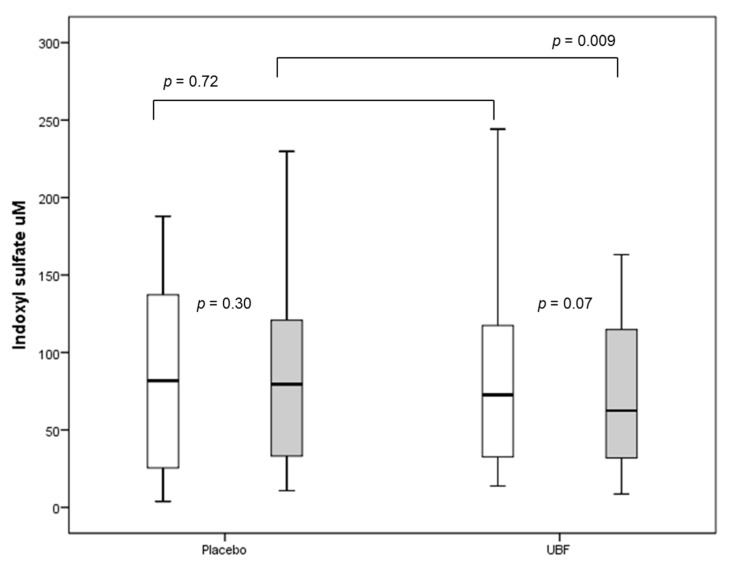
Serum level of total indoxyl sulfate in a subgroup of patients (*n* = 11) undergoing peritoneal dialysis pre (white) and post (gray) the use of placebo and UBF.

Eleven participants (42.3%) were able to take the two sachets during the entire follow-up with adherence greater than 80%. These participants had a median daily intake of placebo of 10.4 g (10–11.6) and UBF of 18.7 g (17.2–19.7) corresponding to about 8.8 g ± 0.6 of resistant starch daily. In this subgroup, 54.5% were women, 54 ± 15 years old, 36.4% had diabetes, 90.9% had residual diuresis, and 90.9% were undergoing nocturnal intermittent peritoneal dialysis (NIPD). These characteristics were not different from the group of participants with adherence less than 80% (*p* > 0.05). Table 4 shows the impact of UBF on uremic toxins in this subgroup. As shown, UBF significantly reduced the serum levels of total IS when compared to placebo (Figure 3). No differences were observed in free serum levels of IS and total and free serum levels of pCS and IAA. During the follow-up, total weekly Kt/V did not change and RRF (mL/min./1.73 m^2^) slightly reduced in the placebo arm (pre-placebo: 5.1 ± 2.4 versus post-placebo: 5.7 ± 2.6, *p* = 0.002). Despite this, the urinary and dialysate excretion of uremic toxins did not differ between the arms during the follow-up (Table 4).

## 4. Discussion

In the present study, we tested the effect of UBF on the serum levels of gut-derived uremic toxins in patients undergoing peritoneal dialysis. We found that supplementation with UBF for 4 weeks did not affect either the serum levels of IS or pCS and IAA.

UBF contains a large amount of dietary fiber (about 56%) with RS representing approximately 87% of the total fiber. The beneficial effect of RS on uremic toxins has not been clearly demonstrated. In HD patients, while a decrease in serum total IS was found in two studies with the administration of 16–18 g/day of RS [34,36], in another, with the same prebiotic and dose, no difference was observed in IS [47].

Despite its importance, adherence to the intervention has not always been carefully assessed or monitored in studies. In the present study, even though strategies were adopted to favor the regular use of two sachets of UBF, some participants had low adherence, and others did not tolerate the proposed dose, which corresponded to 10 g of RS, and were then maintained in the study with their tolerable dose. Separately analyzing the participants who were able to take more than 80% of the proposed dose (8.8 g ± 0.6 of RS daily), serum levels of total IS decreased after UBF compared to placebo. It is important to highlight that the reduction was not due to increased urinary excretion, dialysis removal of IS, or changes in usual dietary intake. This finding suggests that the impact of UBF on the serum IS may be dependent on the dose of UBF and probably the amount of RS.

The type and amount of substrate that reach the human colon are key modulators of bacterial composition and metabolism. An increase in carbohydrates available for fermentation in the colon seems to favor carbohydrates fermentation over nitrogenous compounds, leading to a reduction in specific metabolites such as indole, which is the precursor of IS [25,26]. It has been suggested that the effect of RS on IS is the result of changes in the composition and metabolism of the gut microbiota promoted by the selective growth of certain bacterial species [48]. However, in CKD, there is a paucity of studies in which the impact of RS on the composition of the gut microbiota has been evaluated. In a study with CKD rats fed diets supplemented with RS, the reduction in serum IS was accompanied by changes in gut microbiota composition, markedly by an increase in microbial diversity, in the *Bacteroidetes*-to-*Firmicutes* ratio and in the *Ruminococcus bromii*, which seems to be the keystone species for the degradation of RS [35,49,50]. As far as we know, there is only one study with HD patients that evaluated the impact of RS administration on the fecal microbial profile and the only difference observed was an increase in the *Faecalibacterium prausnitzii* [51], a butyrate-producing species [52,53], which appears to be reduced in individuals with CKD [54] and is frequent and abundant in the gut microbiota of healthy individuals [55]. However, it is important to highlight that this finding was based on the analysis of five specific genera and not on a complete overview of the fecal microbiota composition. Unfortunately, in the present study, the gut microbiome was not assessed.

Regarding the other two gut-derived uremic toxins evaluated (pCS and IAA), no impact of UBF was observed. Similarly, no difference in the serum total pCS was observed in other RS intervention studies with HD patients [34,36]. In healthy individuals, the production of IS and pCS does not seem to be correlated [28], suggesting that the regulation of their production occurs by different factors. In a study with patients undergoing hemodialysis, high pCS levels were associated with distinct gut microbiota composition when compared to high IS levels [56]. Therefore, it is possible that different types of prebiotics are needed to modulate the production of these uremic toxins. While RS seems to impact the generation of IS, other prebiotics appear to be able to reduce the generation of pCS. In HD patients, the daily intake of 20 g of oligofructose-enriched inulin decreased by about 20% serum levels of pCS, but not IS [57]. In another study with 15 g of inulin plus 10 g of pea hull fiber, a similar reduction only in pCS was observed in non-dialysis-dependent CKD patients [58]. In patients in the same stage of the disease, a trend to reduce only serum pCS was found with fructooligosaccharide (FOS) supplementation [59]. Thus, it can be speculated that the combination of specific prebiotics is necessary to modulate the production of these two uremic toxins.

Despite the findings mentioned above, well-designed prebiotic intervention studies in CKD and particularly in PD are scarce. Recently, no changes were also observed in the serum levels of pCS and IS in a well-designed cross-over study in PD, with 10 g of inulin [60]. It is possible that higher doses are required to reduce the serum levels of uremic toxins and the required dose may vary according to the type of prebiotic. In healthy individuals, it was observed that the bifidogenic effect of prebiotics is dose-dependent and the minimum dose required may vary depending on the type of prebiotic. While for FOS it seems the minimum dose is 10 g/day, for inulin, a lower dose seems to be enough (2.5–5 g/day) [61]. As far as we know, there is no study in CKD that evaluated the dose–response effect of prebiotics on the serum level of uremic toxins. In our study, a reduction in serum IS was observed only in the subgroup of participants who consumed approximately 20 g of UBF (10 g of RS)/daily. Although we presume the reduction in serum IS observed in the subgroup was a consequence of RS, we cannot rule out the possibility that other components of the UBF have synergistically contributed to this effect.

In the present study, the use of UBF did not result in changes in the inflammatory markers evaluated (hsCRP, TNF-α, IL-6, and IL-10), nor in the serum levels of LPS (marker of gut permeability). The slight increase observed in IL-6 after UBF does not appear to be an effect of the intervention, since no difference was found between UBF and placebo. In addition, no difference was observed in the levels of IL-6 in the subgroup that showed greater adherence to the intervention. Several factors contribute to the inflammatory state in CKD, including the uremic toxins [62]. Furthermore, it has been suggested that changes in the composition and metabolism of gut microbiota in CKD negatively impact the intestinal permeability, favoring a chronic inflammatory state. The increase in intestinal permeability allows leakage of bacterial components into the bloodstream, which are recognized by the immune system, triggering pro-inflammatory responses [63]. In this context, the modulation of the gut ecosystem also emerges as a possibility to reduce inflammation, both by reducing the production of uremic toxins and by reversing or mitigating the damage in the gut barrier. RS fermentation by gut microbiota produces short-chain fatty acids (SCFA), mainly butyrate [48], which plays an important role in maintaining the integrity of the intestinal barrier, regulating the local immune system [64]. Although there are some limitations, serum levels of LPS have been used as a marker of intestinal permeability in clinical studies. In the current study, the serum levels of LPS found were lower than those of patients on PD in other cohorts [65,66] and similar to the levels reported in healthy individuals [65]. Therefore, it seems unlikely to expect changes in the levels of LPS and consequently, in the inflammatory markers, by this pathway, with the intervention. To date, there is a paucity of studies that have investigated the effects of prebiotics on inflammatory status in the context of CKD. In a study with CKD rats, RS attenuated the inflammation [67]; however, in clinical studies, the results are still discordant. While, in one study, a reduction in some inflammatory markers was observed (TNF-α and IL-6) [68], in another, with the same dose of RS, no difference was found in the levels of hsCRP and IL-6 of HD patients [36].

Some limitations and strengths of the present study should be acknowledged. The loss of a significant number of participants during the follow-up resulted in a relatively small sample size, which may have limited the statistical power for detection of changes in the outcomes. The long duration of the study required in a crossover design may have contributed to the withdrawals. The low tolerability to the UBF may have prevented finding more consistent results. Indeed, a decrease in serum IS was found in the subgroup of participants who ingested the total dose proposed. Although plausible, this subgroup analysis should be viewed with caution due to potential selection bias introduction. The gut microbiota and their metabolites were not assessed, limiting in-depth understanding of the impact of UBF on the composition and metabolism of gut microbiota and consequently, on the uremic toxins. The strengths are the study design and especially, the control of known confounders such as dietary intake (protein and fiber), urinary excretion, and dialysis removal of the uremic toxins.

## 5. Conclusions

In conclusion, we found that the UBF did not affect either the serum levels of IS or pCS and IAA. A reduction in serum total IS was observed, when about 20 g of UBF was used daily. This finding was independent of dietary intake and urinary excretion and dialysis removal of uremic toxins. No effect of UBF was observed in inflammatory markers and intestinal permeability. Our results highlight the difficulty of implementing the regular use of flours with prebiotics in clinical practice, since a large amount of the supplement seems to be required to promote a significant reduction in uremic toxins. This factor should be considered when developing strategies to target the gut microbiota in CKD, since the potential benefits depend on the continuous use of the supplement. Further studies evaluating different doses of prebiotics and especially, the combination of prebiotic types may be required for a better understanding of their effect on the production of gut-derived metabolites and consequently, on the accumulation of uremic toxins in CKD.

## Figures and Tables

**Table 1:** Baseline characteristics of all randomized patients, patients who discontinued the study, and patients who completed the follow up.

Variables	All Patients (*n* = 43)	Patients Who Discontinued the Study (*n* = 17)	Patients Who Completed the Follow Up (*n* = 26)	*p* *
Age (years)	52 ± 18	49 ± 16	55 ± 12	0.16
Male (*n* (%))	23 (53.5)	9 (52.9)	14 (53.8)	0.95
BMI (kg/m^2^)	25.9 ± 4.1	24.7 ± 3.9	26.7 ± 4.1	0.12
GSRS score	29 (24–41)	35 (29–42)	27.5 (21.2–36.5)	0.03
Diabetes (*n* (%))	13 (30)	4 (23.5)	9 (34.6)	0.44
Dialysis vintage (months)	18 (6–42)	18 (11–49)	16 (5–31)	0.22
Residual diuresis (*n* (%))	35 (81.5)	13 (76.5)	22 (84.6)	0.69
RRF (mL/min./1.73 m²)	4.3 ± 3.3	4.5 ± 2.4	5.8 ± 3	0.20
Urine volume (mL/24 h)	1224 ± 543	1105 ± 532	1294 ± 549	0.33
Weekly Kt/V	2.12 ± 0.55	2.0 ± 0.5	2.2 ± 0.6	0.22
Daily ultrafiltration (mL)	824.6 ± 444	759 ± 442	867.5 ± 448	0.44
Laboratory data				
Urea (mg/dL)	124 ± 26	129 ± 27	120 ± 26	0.31
Creatinine (mg/dL)	8.3 (6.5–11.8)	10.3 (7.4–13.1)	7.4 (6–11.6)	0.26
Albumin (g/dL)	3.9 ± 0.34	3.9 ± 0.31	4 ± 0.36	0.48
Hemoglobin (g/dL)	11.6 ± 1.4	11.5 ± 1.7	11.7 ± 1.2	0.60
HbA1C (%)	5.7 (5.3–7)	5.7 (5.2–6)	5.8 (5.3–7.4)	0.52
hsCRP (mg/dL)	0.23 (0.11–0.53)	0.21 (0.1–0.44)	0.24 (0.1–0.59)	0.58
Uremic toxins				
Serum total IS (μmol/L)	79 (49–150)	84 (55–168)	62 (45–133)	0.24
Serum total pCS (μmol/L)	182 ± 97.7	196 ± 96.3	173 ± 99.4	0.46
Serum total IAA (μmol/L)	8.8 (5.6–14.3)	8.8 (5.7–16.4)	8.8 (5.5–13.3)	0.77
Daily dietary intake				
Energy (kcal/kg)	22 ± 6	21 ± 6	23 ± 6	0.50
Protein (g/kg)	0.75 (0.55–0.95)	0.73 (0.53–1.1)	0.76 (0.57–0.9)	0.84
Fiber (g)	9 (6.5–13.5)	8.8 (6.4–13.1)	9 (6.2–14.5)	0.80
Protein:fiber ratio	5.1 (3.4–8.3)	5 (3.9–7.6)	5.8 (2.8–8.7)	0.98
PNA (g/kg)	0.69 ± 0.13	0.7 ± 0.13	0.69 ± 0.13	0.81

Data are presented as mean ± SD or as the median (interquartile range). BMI—body mass index; GSRS—Gastrointestinal Symptom Rating Scale; RRF—residual renal function; HbA1c—glycated hemoglobin A1c; hsCRP—high sensitivity C-reactive protein; IS—indoxyl sulfate; pCS—p-cresyl sulfate; IAA—indole 3-acetic acid; PNA—protein nitrogen appearance. * Patients who discontinued vs. patients who completed the follow-up.

**Table 2:** Laboratory parameters and dietary intake according to UBF and placebo arms during the follow-up of patients undergoing peritoneal dialysis (*n* = 26).

Variables	UBF		Placebo		*p*
	Pre	Post	Pre	Post	
Laboratory data					
Urea (mg/dL)	121 ± 25	126 ± 31	124 ± 24	128 ± 27	0.37
Creatinine (mg/dL)	7.5 (6–11.7)	8.4 (6.2–12) ^a^	8 (6.7–12.4)	8.4 (6.5–12.8) ^b^	0.01
Sodium (mEq/L)	139 ± 2	139 ± 2	139 ± 2	139 ± 2 ^b^	0.02
Potassium (mEq/L)	4.7 (4.3–4.9)	4.8 (4.3–5.2)	4.6 (4.4–5.2)	4.5 (4.3–5.1)	0.60
Ionized calcium (mmol/L)	1.25 ± 0.08	1.24 ± 0.1	1.24 ± 0.08	1.24 ± 0.09	0.93
Phosphorus (mg/dL)	5.8 (4.9–6.5)	5.5 (5.2–6)	5.5 (4.6–5.9)	5.6 (5–6.3)	0.12
Glucose (mg/dL)	85 (79–118)	91 (77–112)	85 (78–104)	91 (83–103)	0.26
Albumin (g/dL)	3.9 ± 0.3	3.9 ± 0.3	3.9 ± 0.4	3.9 ± 0.3	0.93
HbA1C (%)	5.8 (5.5–7.2)	5.7 (5.3–7.7)	5.9 (5.2–8.3)	5.9 (5.3–8.1)	1.0
Urine volume (mL/24 h)	1272 ± 571	1235 ± 569	1211 ± 551	1312 ± 677	0.60
RRF (mL/min./1.73 m²)	5.7 ± 3	5.7 ± 3.3	5.3 ± 2.8	5.9 ± 3.6	0.05
Weekly Kt/V	2.2 ± 0.6	2.1 ± 0.6	2.1 ± 0.5	2.2 ± 0.6	0.17
Inflammatory markers					
hsCRP (mg/dL)	0.25 (0.1–0.5)	0.20 (0.08–0.5)	0.22 (0.09–0.4)	0.26 (0.1–0.4)	0.08
IL-6 (pg/mL)	3.6 (2.2–5.8)	3.8 (2.8–7.3) ^b^	4 (2–7.1)	4.3 (2.5–7.7) ^b^	0.004
IL-10 (pg/mL)	13.3 (9.7–19.3)	13.3 (9.7–19.9)	15.8 (7.5–21.7)	14.1 (9.6–22.6)	0.52
TNF-α (pg/mL)	71.3 ± 28	68.7 ± 21	71.9 ± 27	71.1 ± 21.7	0.74
LPS (EU/mL)	0.07 (0.05–0.1)	0.09 (0.07–0.2)	0.07 (0.05–0.1)	0.1 (0.06–0.1)	0.28
Daily dietary intake					
Energy (kcal/kg)	21.4 ± 7.1	20.6 ± 7.2	20.8 ± 5.2	20.3 ± 6.1	0.44
Protein (g/kg)	0.74 ± 0.2	0.78 ± 0.3	0.73 ± 0.2	0.74 ± 0.2	0.63
Fiber (g)	7 (5.8–12.2)	9 (6.5–14.6)	10 (6.9–15.2)	9.6 (6.9–13.4)	0.25
Protein:fiber ratio	6 (3.6–9.9) ^c^	6.1 (3.6–8.4)	4.7 (3.4–7)	5.7 (3.4–8)	0.002
PNA (g/kg)	0.69 ± 0.13	0.70 ± 0.17	0.69 ± 0.14	0.72 ± 0.16	0.20

Data are presented as mean ± SD or as the median (interquartile range). RRF—residual renal function; hsCRP—high sensitive C-reactive protein; IL-6—interleukin-6; IL-10—interleukin-10; TNF-α—tumor necrosis factor-α; LPS—lipopolysaccharide; PNA—protein nitrogen appearance. ^a^ *p* < 0.05 versus post-placebo; ^b^ *p* < 0.05 versus pre-UBF; ^c^ *p* < 0.05 versus pre-placebo.

**Table 3:** Uremic toxins according to UBF and placebo arms during the follow-up of patients undergoing peritoneal dialysis (*n* = 26).

Variables	UBF		Placebo		*p*
	Pre	Post	Pre	Post	
Serum total uremic toxins (μmol/L)					
IS	67 (35–141)	63 (35–139)	59 (37–137.5)	72.7 (32.5–136)	0.70
pCS	153.5 (88–283)	171.5 (131–263)	149.5 (95–235)	164 (108–242)	0.70
IAA	8.7 (5.7–13.4)	9 (6–15)	9.7 (5.3–13)	8 (5.8–13.2)	0.74
Serum free uremic toxins (μmol/L)					
IS	1.4 (0.6–4.3)	1.6 (0.7–3.5)	1.7 (0.8–4.2)	1.5 (0.7–3.1)	0.95
pCS	1.8 (0.9–4.7)	2.2 (1–5.5)	2.3 (1–5)	2.3 (1.1–4.6)	0.24
IAA	0.60 (0.2–1.1)	0.65 (0.3–1)	0.68 (0.25–1)	0.62 (0.3–0.8)	0.29
Urinary total uremic toxins (μmol/24 h)					
IS	200 (130–290)	199 (64–263)	210 (87–273)	180 (105–285)	0.36
pCS	97 (30–184)	100 (48–286)	117 (49–240)	97 (39–222)	0.25
IAA	5.7 (2.9–8.6)	5.2 (2.7–11.3)	3.6 (2.5–9.3)	3.9 (2.3–7.6)	0.55
Dialysate total uremic toxins (μmol/24 h)					
IS	30 (14–78)	29 (15–76)	33.4 (19–68)	28.4 (19–88)	0.45
pCS	24 (10–139)	28 (12–88)	30.4 (10–98)	25 (12–85)	0.90
IAA	8.3 (5–18)	10 (5–19)	11.4 (6–19)	11 (6–15)	0.82

Data are presented as mean ± SD or as the median (interquartile range). IS—indoxyl sulfate; pCS—p-cresyl sulfate; IAA—indole 3-acetic acid.

**Table 4:** Uremic toxins in adherent patients according to UBF and placebo arms during the follow-up of patients undergoing peritoneal dialysis (*n* = 11).

Variables	UBF		Placebo		*p*
	Pre	Post	Pre	Post	
Serum total uremic toxins (μmol/L)					
IS	73 (30–136)	62.5 (31–133) ^a,b^	82 (23–163)	79.5 (31–142)	0.001
pCS	136 (82–240)	144 (75–160)	124 (90–226)	144 (74–175)	0.95
IAA	6.5 (5–10)	8.3 (5.8–10.6)	7.6 (4.9–11.1)	7.5 (5.8–10)	0.88
Serum free uremic toxins (μmol/L)					
IS	0.98 (0.6–2.5)	1.0 (0.5–2.2)	0.86 (0.4–3.2)	1.1 (0.5–1.9)	0.24
pCS	1.1 (0.6–2)	1.3 (0.8–1.6)	1.0 (0.9–3)	1.4 (0.6–2.4)	0.54
IAA	0.4 (0.2–0.7)	0.5 (0.2–0.7)	0.3 (0.2–0.8)	0.4 (0.3–0.8)	0.62
Urinary total uremic toxins (μmol/24 h)					
IS	192 (109–240)	176 (49–226)	154 (56–220)	160 (95–238)	0.05
pCS	82 (19–201)	85 (14–232)	60 (25–211)	79 (31–190)	0.76
IAA	4.4 (2.9–7.1)	3.4 (2.7–6)	2.8 (2.2–6.1)	3.1 (2–4.1)	0.14
Dialysate total uremic toxins (μmol/24 h)					
IS	19.7 (14.7–66.1)	19.6 (8.4–33.2)	29.8 (10.3–67.5)	27.9 (11–51)	0.27
pCS	23.8 (5.6–66.7)	13.3 (7.5–51.8)	14.9 (3.5–34.3)	22.5 (2.8–40)	0.30
IAA	6.3 (4.1–15.4)	6.1 (3.9–12.2)	5.9 (4.2–14.1)	9.6 (3.9–13.6)	0.40

Data are presented as mean ± SD or as the median (interquartile range). IS—indoxyl sulfate; pCS—p-cresyl sulfate; IAA—indole 3-acetic acid. Adherent patients—consumption ≥ 80% of the sachets offered. ^a^ *p* < 0.05 versus post-placebo; ^b^ *p* < 0.05 versus pre-placebo.

## Data Availability

The data underlying this article will be shared upon request to the corresponding author.
